# Maximum flow-based resilience analysis: From component to system

**DOI:** 10.1371/journal.pone.0177668

**Published:** 2017-05-17

**Authors:** Chong Jin, Ruiying Li, Rui Kang

**Affiliations:** 1 School of Reliability and Systems Engineering, Beihang University, Beijing, China; 2 Science and Technology on Reliability and Environmental Engineering Laboratory, Beijing, China; US Army Engineer Research and Development Center, UNITED STATES

## Abstract

Resilience, the ability to withstand disruptions and recover quickly, must be considered during system design because any disruption of the system may cause considerable loss, including economic and societal. This work develops analytic maximum flow-based resilience models for series and parallel systems using Zobel’s resilience measure. The two analytic models can be used to evaluate quantitatively and compare the resilience of the systems with the corresponding performance structures. For systems with identical components, the resilience of the parallel system increases with increasing number of components, while the resilience remains constant in the series system. A Monte Carlo-based simulation method is also provided to verify the correctness of our analytic resilience models and to analyze the resilience of networked systems based on that of components. A road network example is used to illustrate the analysis process, and the resilience comparison among networks with different topologies but the same components indicates that a system with redundant performance is usually more resilient than one without redundant performance. However, not all redundant capacities of components can improve the system resilience, the effectiveness of the capacity redundancy depends on where the redundant capacity is located.

## Introduction

Modern society is built on adaptive and intelligent infrastructure systems that deliver energy and information to support productivity, water to meet basic needs, and transportation to connect communities. Currently, infrastructure systems are becoming increasingly smarter in normal operation and use. However, the infrastructure systems are vulnerable to many natural disasters and man-made attacks that threaten the services they provide, and the performance degradation may cause considerable financial loss. For example, the “9/11” terrorist attack on the World Trade Center caused a power outage in New York in 2001, and it took several weeks to restore the entire urban electricity supply [1]; on August 14, 2003, some overloaded transmission lines hit overgrown trees in Ohio, which resulted in a large blackout and an estimated $6 billion impact on the USA and Canadian economies [2]; and, in 2015, multiple airlines suffered computer failures, such as United Airlines, Delta and British Airways, which caused widespread delays or even flight cancellations [3]. In addition, the 2009 L’Aquila earthquake in Italy and the 2011 Tohoku earthquake in Japan also exemplified the vulnerability of our modern, highly complex infrastructure systems. To face so many surprising combinations of events and more extreme stressors, building resilience becomes the best decision for large, complex infrastructure systems [4]. Park et al. [5] described resilience analysis as complementary to risk analysis with important implications for the adaptive management of complex systems.

Actually, the concept of resilience was introduced more than 40 years ago in ecology by Holling [6] and was then extended to organization and management [7, 8], economy [9, 10], psychology [11, 12], and other engineering fields [13–18]. Along with its increasing appearance in calls for research proposals and scientific databases, the term resilience has become increasingly popular in the last decade [19], and some government agencies have increasingly emphasized resilience planning for infrastructure systems [20–22]. Correspondingly, resilience has been used in various practical fields to evaluate the ability of targeted systems to respond to disruptions that threaten their normal operation. More background on current developments in the fields can be found in the recently published IRGC (International Risk Governance Council) Resource Guide on Resilience (https://www.irgc.org/irgc-resource-guide-on-resilience/). For example, the RESOLUTE project (http://www.resolute-eu.org) of Europe aims at enhancing the resilience of urban transport systems (UTS), where resilience is considered as a useful management paradigm, within which adaptability capacities are considered paramount [23]; the project of 100 Resilient Cities (100RC, http://www.100resilientcities.org/), pioneered by the Rockefeller Foundation, aims at not only helping individual cities become more resilient but also building a global resilience practice among governments, private sectors, and individual citizens [24]. Based on the original definition of resilience put forward by Holling in ecology, recent research [25–27] and guidance documents [28, 29] provided several other definitions for resilience. For example, “Resilience is the ability to prepare and plan for, absorb, recover from, and more successfully adapt to adverse events [28]”; “Resilience includes the ability to prepare for and adapt to changing conditions and withstand and recover rapidly from disruptions [29]”. Although resilience has been researched for more than forty years, a universally accepted definition of resilience has not yet been unified. Proag [30] and Hosseini et al. [31] summarized that most research about system resilience focuses on the ability of systems to withstand disruptions and recover quickly.

To improve the resilience of infrastructure systems, it is critical to understand how resilience can be measured. Many recent attempts to quantify the resilience of technological systems were based on performance degradation and recovery from a single disruption. The most famous one is the “resilience triangle”, which originates from the seismic disaster research by the MCEER (Multidisciplinary Center for Extreme Event Research) group [32]. They defined “resilience loss” as the size of the expected degradation in normalized quality over time during recovery after disruption. Reed et al. [33] expanded this measure and defined system resilience as the ratio of the integration of normalized quality function over the recovery time to the length of the period, reflecting the average normalized quality of the system after a disruption. Cimellaro et al. [34] modified the definition of resilience proposed by Reed et al. [33] and focused on the change in system quality over the control time (usually the life cycle, life span of the system, etc.) instead of the recovery time. However, considering that the recovery time or control time of different systems differs, it is difficult to use the above measures for system resilience comparison, and real-time performance data cannot be obtained in the system design stage. A simplified geometry-based method was provided by Zobel [35] to predict system resilience, in which the recovery rate of system performance is recognized as a constant. Ouyang and Dueñas-Osorio [36] also considered the consistency of the time scale and developed a time-dependent resilience measure. This measure is built on the system performance monitoring data within a period from 0 to *T*, quantified as the ratio of the area between the real performance curve and the time axis to the area between the target performance curve and the time axis during the period. Henry and Ramirez-Marquez [37] proposed a time-dependent resilience metric, which is defined as the ratio of system recovery at time *t* to the loss it suffered and can be used to describe the dynamic recovery behavior of the system after the disruption. To reflect the uncertainty of the system resilience, the MCEER group [38] also defined a probabilistic resilience that measures the probability that the expectation of the resilience loss will exceed the performance limit state, but the specific expression is not given. Ouyang et al. also developed an expected annual resilience under multi-disruption events based on their time-dependent resilience measure in [36] to reflect the stochastic characteristics of system behavior, and the occurrence rates of different hazard types are integrated into an expected annual resilience metric.

Due to the interdependency of nodes and links, networked systems generally tend to be less robust and more likely to be vulnerable to perturbations. Murray-Tuite and Mahmassani [39] combined the availability of alternate paths, excess capacity, and travel time to describe the disruption of transportation networks. Morlok and Chang [40] studied the capacity flexibility through changes in demand and traffic patterns. Sterbenz et al. [41] used the metrics that quantify service requirements and operational state to detect and quantify resilience. Henry and Ramirez-Marquez [37] used three figures of merit—i.e., the shortest path length, the maximum flow and the overall health—to quantify the resilience of the transportation network. Bhatia et al. [42] used the percentage of the active stations in the networked system to measure the resilience, and both the hazard responses and recovery strategies were compared using the Indian Railways Network as an example. In another study, Ganin et al. [43] proposed a quantitative measure of resilience based on the evaluation of critical functionality of a networked system over time, in which the proportion of active nodes in a network was considered as a measure of performance, and illustrated how parameterizations for features such as redundancy, node recovery time, and available backup supply could be tuned to obtain a desired resilience state. Among the above research on network resilience, maximum flow is one of the most frequently used performance measures in network resilience analysis [37, 39]. Similarly, Agarwal et al. [44] defined network resilience in terms of the expected maximum flow measure for a future network with dynamic restoration capability and provided a unified framework to identify vulnerable points for the WDM network, which can significantly improve the network resilience if a protection plan is taken at these vulnerable points. Omer et al. [45] defined network resiliency as the ratio of the value delivery of the system after a disruption to the value delivery of the system before a disruption, and they showed that rerouting and redundant capacities will improve network resilience. Baroud et al. [46] and Pant et al. [47] used commodity flow to measure the network performance in their practical inland waterway network and inland port and provided a resilience optimization method. Como et al. [48, 49] studied the robustness of dynamical flow networks by evaluating the network’s weak and strong resilience and showed that the weak resilience of the network is equal to the min-cut capacity and is independent of the local information constraint and the initial flow, while the strong resilience is equal to the minimum node residual capacity and is sensitive to local information constraint. As a consequence, we choose maximum flow as the system performance metric and discuss the maximum flow-based resilience throughout the paper.

Using the above measures of resilience, pioneers analyzed several systems based on real data and simulated data. However, only a few studies considered how the resilience of components affects that of the system. Reed et al. [33] found that the resilience of an *n*–system infrastructure is a function of the resilience of the individual subsystem—i.e., $R_{S}=g\left(R_{1},R_{2},\ldots ,R_{n}\right)$—but the specific formula was not discussed. Filippini and Silva [50] built a functional relationship between components and system resilience. Their resilience is defined as the number of active nodes in the system, so the system resilience can be calculated by adding all the states (defined as a binary variable) of components together. However, Filippini and Silva’s functional relationship is not applicable to maximum flow-based resilience because the relationship between the maximum flow of the system and the capacity of components is quite different. In addition, Diao et al. [51] designed a global resilience analysis (GRA) framework to assess the whole-system resilience of engineering systems. By identifying the failure modes of the system, determining an appropriate number of failure scenarios, simulating failure mode strains under increasing stress magnitude, the system resilience is calculated. The number of failure scenarios is determined by considering different number of components disrupted by random and target scenarios. This paper provides us a system thinking of measuring the whole system resilience. To date, we are aware of no study that has put forth an analytic model of system resilience based on the component performance and the system structure, and our paper is among the first to discuss this problem. Based on the maximum flow, two general and practical analytic resilience models for series and parallel systems are derived and are applicable to systems of any size. According to the same process to calculate the resilience of series and parallel systems, a simulation method based on Monte Carlo is proposed to help stakeholders identify how the damage of different components affects the resilience of the networked system, compare the system resilience for different topological structures and capacity redundancy, and determine the component whose capacity increase will improve system resilience.

## Methodology

### Problem description

**Fig 1:**
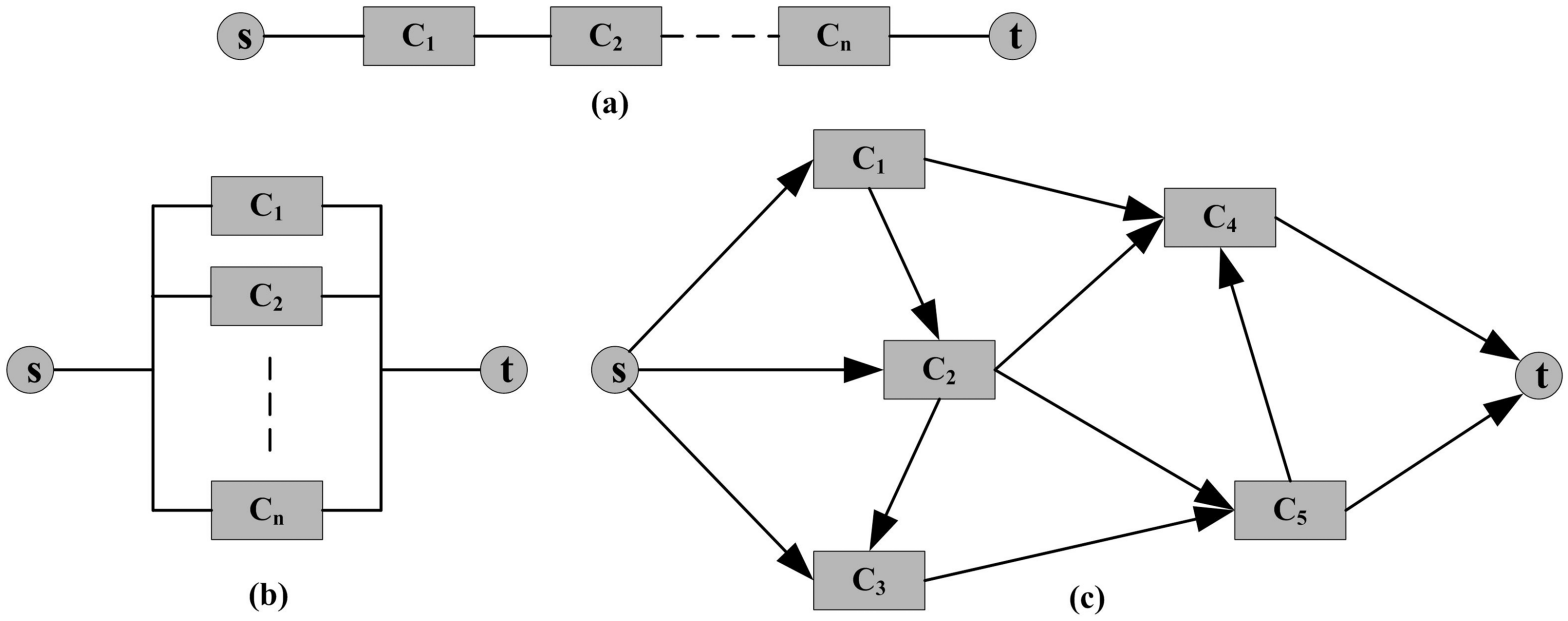
The block “diagram” of different system structures. **(a)** series, **(b)** parallel and **(c)** networked systems.

In our paper, resilience is defined as the ability of systems to withstand disruptions and recover quickly, and Zobel’s resilience measure is used to discuss systems with series, parallel and networked structures as illustrated in Fig 1. Disruptions can occur on any components of the system. Once the component is disrupted, its performance may decline, and such loss may even propagate to the system. The systems with different structures may behave differently under the same disruption scenario because the capacity redundancy of systems with different structures varies.

One can find that the component performance contributes to the system differently for different types of performance. For example, the maximum flow of a series system is determined by the minimum capacity of all its components, its transmission delay is computed by adding all the delays on the components, and its error rate is calculated by multiplying all the error rates of components together. According to the above literature review for performance metrics, maximum flow is chosen as the key performance index in our study. As one of the most representative performance metrics of a system, maximum flow mainly depends on the component capacity and the system structure.

As is well known, the disruption the system suffers, the performance degradation and the recovery time are typical random behaviors. To discuss how component resilience affects the system, the following assumptions are considered in our paper:

1. disruption: only one disruption can occur at a time, each disruption affects only one component of the system, and the probability that the disruption occurs on the *i*^th^ component is *q*_*i*_;
2. performance degradation: the capacity degradation of components follows a discrete distribution (for component *i* with initial capacity *C*_*i*_, the possible values to which its capacity can be degraded are *C*_*i*,1_, *C*_*i*,2_, …, *C*_*i*, *m*_*i*__, and the probability of each value is $p_{i,k}=P\left\{C_{i}^{*}=C_{i,k}\right\}$, where $C_{i}^{*}$ is its capacity after the performance degradation);
3. recovery: the recovery time of components follows a lognormal distribution (for component *i*, the recovery time $t_{i}∼\ln N\left(\mu_{i},\sigma_{i}^{2}\right)$).

Note that Assumption 1 is the most general assumption used in the previous analysis of system reliability [52–54] to simplify the problem. In their work, failures among components are independent, and no common failure cause exists. Systems that may suffer common cause disruptions will be discussed in our future work. Assumption 2 uses discrete distributions for component capacity degradation because the capacity of components in the stochastic flow network is usually supposed to follow a discrete distribution [55, 56]. The recovery time of a component depends not only on the severity of the disruption but also on its supportability, which determines the time that the work force, equipment, spares, etc. are ready for use. In most situations, the time consumed by waiting for such resources is much longer than the time used by the repair process itself. Zobel [35] also noted that whether the resources can be quickly accessed largely affects the recovery time of the system. Thus, in our paper, the recovery time of a component is assumed to be independent of the severity of the disruption. This assumption is widely used in resilience analysis. For example, Ouyang et al. [57] assumed that the variables that constitute restoration time satisfy a uniform distribution and an exponential distribution when analyzing the resilience of infrastructure with their multi-stage framework; Barker et al. [58] and Baroud et al. [46] considered that the recovery time of arcs follows a uniform distribution in a given interval in their study on resilience-based component importance measures. Moreover, a lognormal distribution is the most widely used distribution for system repair time [59–61], and according to the analysis in [62, 63], the incident duration (including incident detection and recovery time) of traffic systems also follows a lognormal distribution. Consequently, we choose a lognormal distribution to describe the recovery time in Assumption 3.

### Zobel’s resilience measure

**Fig 2:**
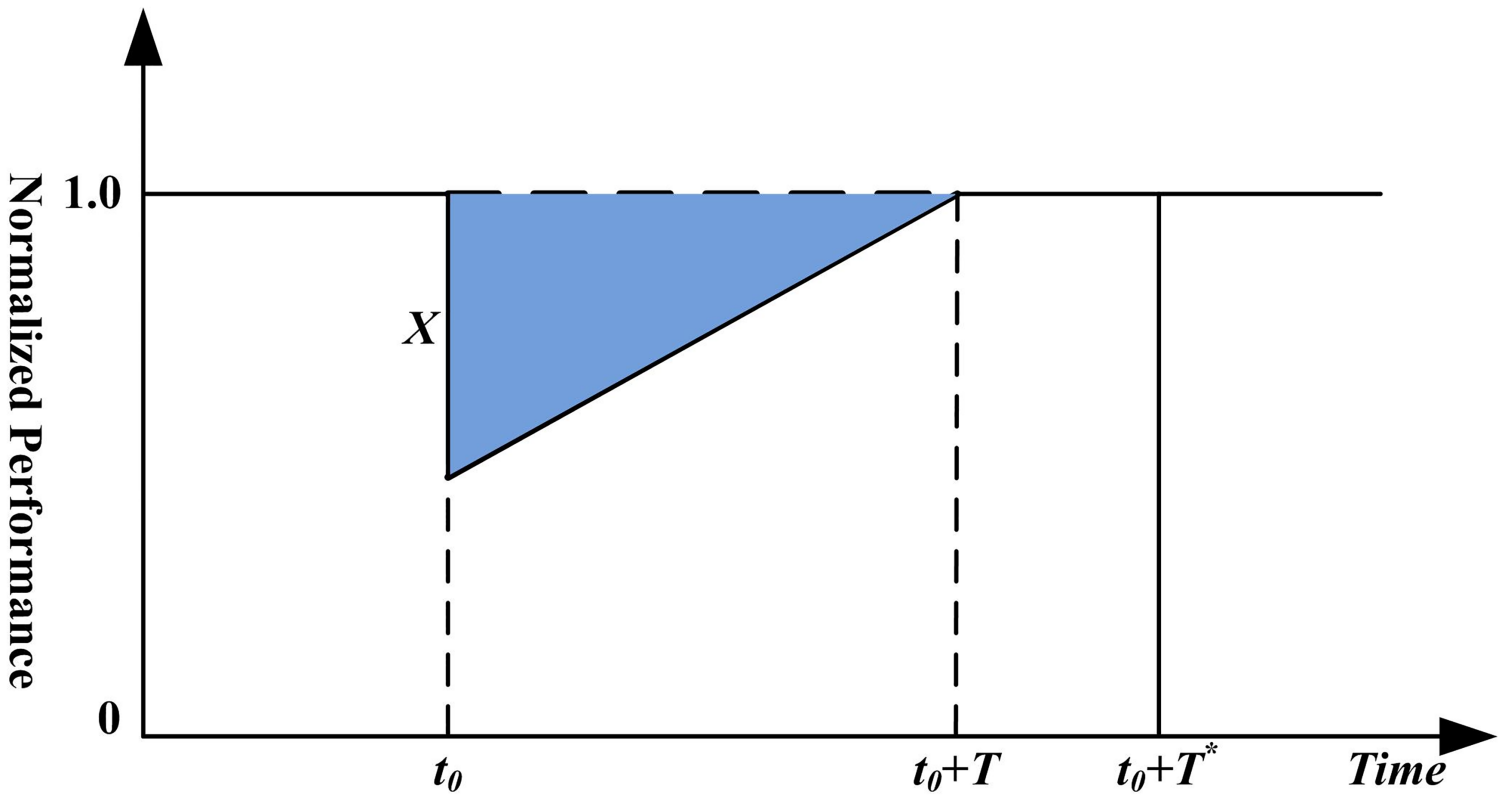
Predicted resilience triangle.

As mentioned above, the “Resilience Triangle” is the most commonly used measure, and Zobel [35] defined the predicted resilience of a system against future disruptions. Because system performance after disruption cannot be obtained during the system design, Zobel assumed that the system recovers at a constant rate, and two parameters, the initial normalized performance loss *X* and the subsequent recovery time *T*, are used to determine the linear recovery process. As shown in Fig 2, a disruption occurs at time *t*_0_, and the predicted resilience of the system is determined by subtracting the area of the triangle from the larger rectangular area and then representing the result as a percentage of that larger area, i.e.,

$$\begin{array}{c} R\left(X,T\right)=\frac{T^{*}-XT/2}{T^{*}}=1-\frac{XT}{2T^{*}}, \end{array}$$ (1)

where T* is a strict upper bound on the set of possible values for *T*. One can see that the area of the triangle is the amount of time-varying loss suffered by the system after a particular disruption, and the resilience is the average performance of the system after the disruption in a T* time interval.

In our problem, by dividing the current capacity of the component/maximum flow of the system by the initial one, the two parameters can be normalized. The capacity-based resilience of component *i* can be calculated as

$$\begin{array}{c} R_{i}=1-\frac{\left(1-\frac{C_{i}^{*}}{C_{i}}\right)t_{i}}{2T^{*}}=1-\frac{\left(C_{i}-C_{i}^{*}\right)t_{i}}{2C_{i}T^{*}}. \end{array}$$ (2)

According to the assumptions in our problem description, the capacity degradation of components follows discrete distributions, and their recovery time follows a lognormal distribution. Hence, the expected capacity-based resilience of component *i* can be computed as

$$\begin{array}{c} E\left({\mathbb{R}}_{i}\right)=1-\frac{\left[1-\frac{\sum_{k=1}^{m_{i}}\left(p_{i,k}C_{i,k}\right)}{C_{i}}\right]e^{\mu_{i}+\frac{1}{2}\sigma_{i}^{2}}}{2T^{*}}=1-\frac{\left[C_{i}-\sum_{k=1}^{m_{i}}\left(p_{i,k}C_{i,k}\right)\right]e^{\mu_{i}+\frac{1}{2}\sigma_{i}^{2}}}{2C_{i}T^{*}}, \end{array}$$ (3)

where *E*[⋅] is the expected value.

### Resilience model for series systems

**Fig 3:**
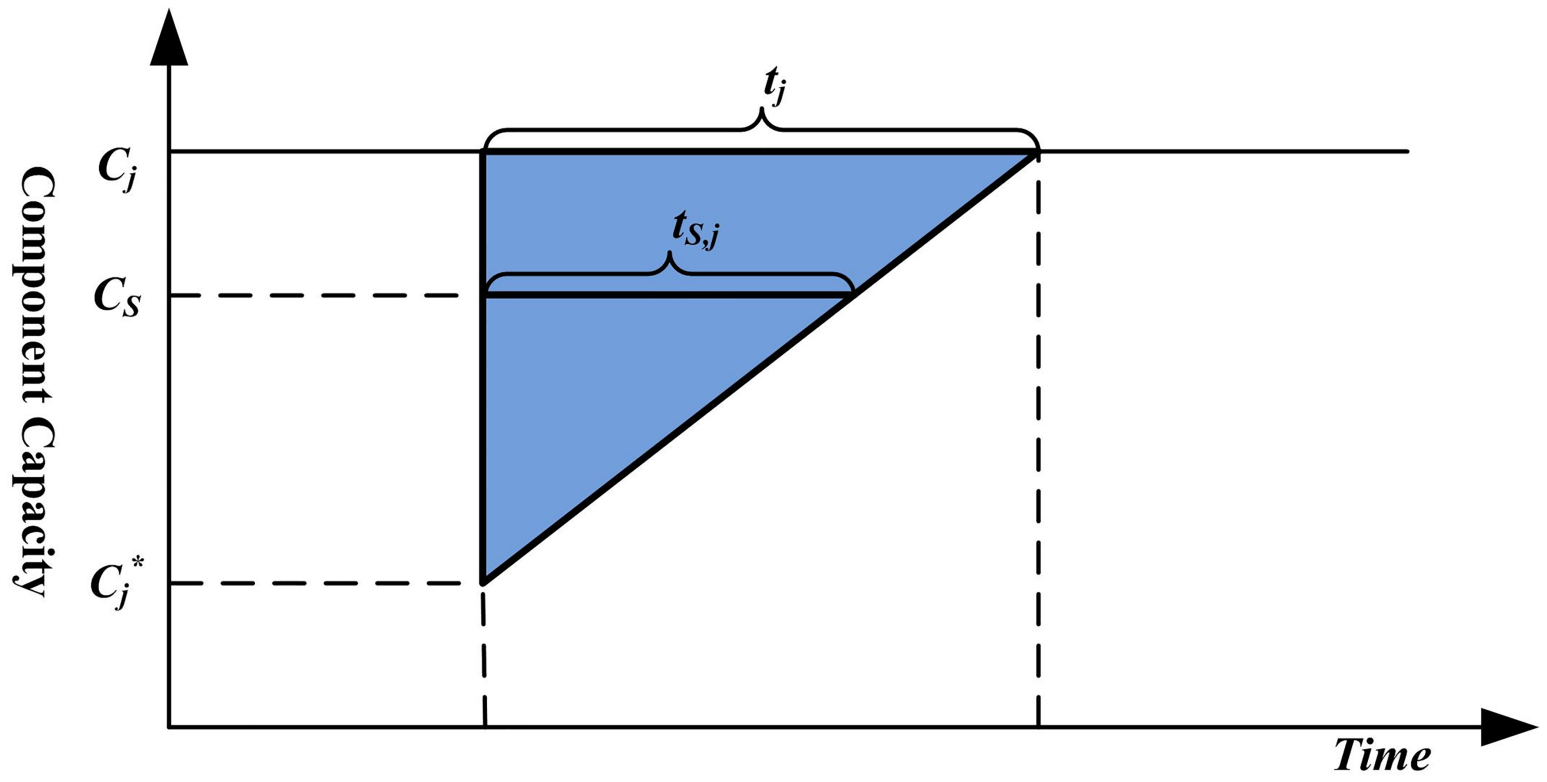
The recovery time calculation for series systems based on the similar triangle principle.

As shown in Fig 1(a), for a system with a series connection, the maximum flow equals the minimum capacity of all its components. The initial maximum flow *C*_*S*_ of the system is determined by the capacity of components as $C_{S}=\min_{i=1,2,\ldots ,n}\left\{C_{i}\right\}$. When a disruption occurs on component *j*, its capacity drops to $C_{j}^{*}$ after the disruption, and the corresponding maximum flow of the system can be calculated as $\min_{i\neq j}\left\{C_{i},C_{j}^{*}\right\}$. For series systems, the degradation of component capacity does not always disrupt the system maximum flow, which reflects that the system can withstand such disruption. The system maximum flow degrades only when the capacity of any component declines to a value below the initial system maximum flow *C*_*S*_. Once the system maximum flow degrades, it will recover to the normal level as long as the capacity of the degraded component recovers to *C*_*S*_. Using the similar triangle principle as illustrated in Fig 3, the recovery time of the series system caused by the disruption on component *j* (from the time the disruption occurs to the time the maximum flow of the system recovers) can be calculated as

$$\begin{array}{c} t_{S,j}=\frac{C_{S}-C_{j}^{*}}{C_{j}-C_{j}^{*}}t_{j},\;\left(C_{j}^{*}<C_{S}\right), \end{array}$$ (4)

where *t*_*j*_ is the recovery time of disrupted component *j*. The resilience of the series system under the degradation of component *j* can be computed as

$$\begin{array}{c} R_{S,j}=1-\frac{\left(1-\frac{C_{j}^{*}}{C_{S}}\right)t_{S,j}}{2T^{*}}=1-\frac{{\left(C_{S}-C_{j}^{*}\right)}^{2}t_{j}}{2C_{S}\left(C_{j}-C_{j}^{*}\right)T^{*}},\;\left(C_{j}^{*}<C_{S}\right). \end{array}$$ (5)

In a series system with *n* components, given the disruption probability of the *i*^th^ component as *q*_*i*_, we can obtain the expected maximum flow-based system resilience as

$$\begin{array}{c} \begin{array}{c} E\left({\mathbb{R}}_{S}\right)=\sum_{i=1}^{n}q_{i}E\left({\mathbb{R}}_{S,i}\right)=1-\frac{\sum_{i=1}^{n}q_{i}\left[\sum_{\text{if }C_{i,h}<C_{S}}p_{i,h}\left(\frac{{\left(C_{S}-C_{i,h}\right)}^{2}}{C_{i}-C_{i,h}}\right)\right]e^{\mu_{i}+\frac{1}{2}\sigma_{i}^{2}}}{2C_{S}T^{*}}. \end{array} \end{array}$$ (6)

### Resilience model for parallel systems

See Fig 1(b), for a system with parallel connection, the maximum flow equals the sum of the capacities of all its components. The initial maximum flow *C*_*P*_ of the system can be calculated as $C_{P}=\sum_{i=1}^{n}C_{i}$. When a disruption occurs on component *j* and its capacity declines to $C_{j}^{*}$, the corresponding maximum flow of the system can be computed by $C_{j}^{*}+\sum_{i\neq j}C_{i}$. For parallel systems, capacity degradation on any component will lead to a loss of system maximum flow, and only when the degraded component restores fully will the performance of the system recover to its initial level. Because the recovery time of the system equals that of the disrupted component, the resilience of the parallel system under the degradation of component *j* can be calculated as

$$\begin{array}{c} {\mathbb{R}}_{P,j}=1-\frac{\left(1-\frac{C_{j}^{*}+\sum_{i\neq j}C_{i}}{C_{P}}\right)t_{j}}{2T^{*}}=1-\frac{\left(C_{j}-C_{j}^{*}\right)t_{j}}{2C_{P}T^{*}}. \end{array}$$ (7)

Similarly, in a parallel system with *n* components, given the disruption probability of the *i*^th^ component as *q*_*i*_, we can obtain the expected maximum flow-based system resilience as

$$\begin{array}{c} E\left({\mathbb{R}}_{P}\right)=\sum_{i=1}^{n}q_{i}E\left({\mathbb{R}}_{P,i}\right)=1-\frac{\sum_{i=1}^{n}q_{i}\left(C_{i}-\sum_{k=1}^{m_{i}}p_{i,k}C_{i,k}\right)e^{\mu_{i}+\frac{1}{2}\sigma_{i}^{2}}}{2C_{P}T^{*}}. \end{array}$$ (8)

### Resilience analysis for networked systems

The above two subsections provide analytic maximum flow-based resilience models for series and parallel systems. However, networked systems as shown in Fig 1(c) are more common in practice. Here, the maximum flow of the network is calculated using the algorithm derived from Edmonds and Karp [64]. Both nodes and links are the components of the system. To simplify the problem, we consider only the capacity and disruption of links in this paper. For nodes that may also suffer disruptions, they can be converted to links. It is not easy to obtain the analytic resilience model for networked systems because no explicit function exists between component capacity and network maximum flow. Hence, we use Monte Carlo simulation to explore the network resilience and illustrate how the damage of different components affects the resilience of the networked system. The steps of the simulation are as follows.

1. Calculate the initial maximum flow from source to terminal, *C*_*N*_.
2. According to the disruption probability of each component *q*_*i*_, use the Monte Carlo-based sampling method to determine the component *j* that suffers a disruption.
3. Obtain the remaining capacity $C_{j}^{*}$ after the disruption and recovery time *t*_*j*_ for component *j* by randomly sampling according to their corresponding distributions.
4. Apply Zobel’s resilience measure to calculate the capacity-based resilience of component *j* as Eq (2).
5. Calculate the system maximum flow after the disruption, i.e., *C*_*N*_*j*__.
6. Determine whether all degraded levels of component $C_{j}^{h}$ will cause the degradation of the system maximum flow. If yes, *T*_*N*, *j*_ = *t*_*j*_; if not, find the lowest capacity that component *j* needs to support the initial maximum flow of the system, denoted as $C_{j}^{h}$, where *h* is the capacity level number in the discrete distribution of component *j*. Calculate the recovery time of the system based on that of component *j* using the similar triangle principle, i.e., $T_{N,j}=\frac{C_{j}^{h}-C_{j}^{*}}{C_{j}-C_{j}^{*}}t_{j}$.
7. Compute the maximum flow-based system resilience under the k*^th^* disruption as $R_{N,k}=1-\frac{(C_{N}-C_{N_{j}})T_{N,j}}{2C_{N}T^{*}}$.
8. To consider the randomness of the disruption, capacity degradation and recovery time, repeat Step 2 to Step 7 for a chosen number of iterations, *M*.
9. Finally, calculate the empirical system resilience using the above system resilience values under different disruptions as $\bar{R_{N}}=\frac{\sum_{k=1}^{M}R_{N,k}}{M}$.

According to the simulation results, we can analyze the simulation error. It is well known that the arithmetic average of the samples obtained by Monte Carlo simulation from one population follows a normal distribution with mean *μ* and variance $\frac{\sigma^{2}}{N}$ for large sample size *N*. Given the two-sided confidence level as (1 − *α*) (eg., 1 − *α* = 95%), the simulation deviation can be calculated as

$$\begin{array}{c} \varepsilon =\frac{z_{\alpha /2}S}{\sqrt{N}}, \end{array}$$ (9)

where *z*_*α*/2_ is the $100(1-\frac{\alpha }{2})\text{th}$ percentile of the standard normal distribution, and *S* is the standard deviation (sd) of all system resilience values. According to our simulation method, the computation time complexity can be calculated as *O*(*max* − *flow*) + *N* × (*O*(*rand*(*e*)) + *O*(*sort*(*e*)) + 2 × *O*(*rand*) + *O*(*max* − *flow*)), where *O*(*max* − *flow*), *O*(*rand*) and *O*(*sort*) are the time complexities of the maximum flow algorithm, the random number generator and the sorting algorithm, respectively; *N* is the number of simulation iterations; and *e* is the number of edges. The Edmonds and Karp algorithm, Mersenne twister algorithm and quick sort algorithm are used as the maximum flow algorithm, the random number generator and the sorting algorithm in our paper, and their time complexities are *O*(*n* × *e*^2^), *O*(*e*) and *O*(*e*^2^), respectively, where *n* is the number of nodes. Thus, the computation time complexity of our simulation method can be calculated as *O*(*N* × (*n* × *e*^2^)), which is a P-hard problem that can be used for large-scale networks.

## Results and discussion

### Series and parallel systems

#### Illustrated example

To illustrate and verify the analytic resilience models, two systems with 4 components are used as examples. The series and parallel networks have widespread applications in daily life. For example, the end-to-end data transmission on a network with a virtual link is a typical series connection, and a two-layered supply chain network with multiple suppliers and one manufacturer can be considered as a parallel network. The parameter data of components are shown in Table 1, where the capacity degradation and the recovery time of components follow a discrete distribution and a lognormal distribution, respectively. The strict upper bound of the recovery time T* is 20 time units.

**Table 1:** The parameter data of the case.

Component	Initial capacity	Disruption probability	Remaining capacity	Probability	Recovery time
Com_1_	4	0.4	0*	0.1	*t*_1_ ∼ *lnN*(0.3, 0.5^2^)
			1	0.2	
			2	0.3	
			3	0.4	
Com_2_	5	0.3	0	0.1	*t*_2_ ∼ *lnN*(0.8, 0.5^2^)
			2	0.15	
			3	0.25	
			4	0.5	
Com_3_	7	0.2	1	0.15	*t*_3_ ∼ *lnN*(1.2, 0.5^2^)
			2	0.1	
			4	0.3	
			5	0.25	
			6	0.2	
Com_4_	10	0.1	2	0.1	*t*_4_ ∼ *lnN*(1.5, 0.5^2^)
			3	0.15	
			5	0.25	
			6	0.2	
			8	0.3	

* *Pr*{the remaining capacity of Com_1_ is 0} = 0.1.

#### Analytic analysis and simulation verification

Using our analytic resilience models, the expected maximum flow-based system resilience is calculated as column 2 in Table 2. To verify the two analytic models, Monte Carlo simulation is used again. After 10^5^ simulation iterations, the empirical maximum flow-based system resilience is computed in column 3. With the simulation results, the simulation error can be calculated using Eq (9), and the results are illustrated in column 4.

**Table 2:** Comparison of system resilience results using analytic and simulation methods.

Structure	Analytic result (E(R))	Simulation result ($\bar{R}$)	Simulation error (*ε*)	Absolute error ($|E\left(R\right)-\bar{R}|$)
Series system	0.987641	0.987625	1.02 × 10^−4^	1.60 × 10^−5^
Parallel system	0.993037	0.993048	5.57 × 10^−5^	1.10 × 10^−5^

According to Eq (3), the resilience of the components is determined by the performance degradation (*X*), the recovery time (*T*), and the upper bound of the recovery time (T*). In our case study, T* = 20, the expected values of the recovery time for Com_1_-Com_4_ are 1.5296, 2.5219, 3.7622 and 5.0784, and the expected resilience values for the four components are 0.9809, 0.9754, 0.9597 and 0.9429, respectively. The differences among components are small because the large T* makes the resilience results very high. Similarly, although the difference between the resilience of the series and parallel system is not large, the average system performance in the early disruption and the whole performance recovery process are quite different.

The absolute errors between the analytic and simulation methods are illustrated in column 5 of Table 2, which are obviously less than the expected simulation errors. Using back-to-back verification, the results indicate the correctness of both our analytic resilience models for series/parallel systems and the simulation method based on Monte Carlo. Moreover, the sequence of the components in the series and parallel networks does not affect our resilience calculation results. With the same components suffered the same disruptions, the system resilience is the same (see our analytic resilience models in Eqs (5)–(8)).

#### Resilience analysis and discussion

**(1) Resilience analysis under different structures**

**Fig 4:**
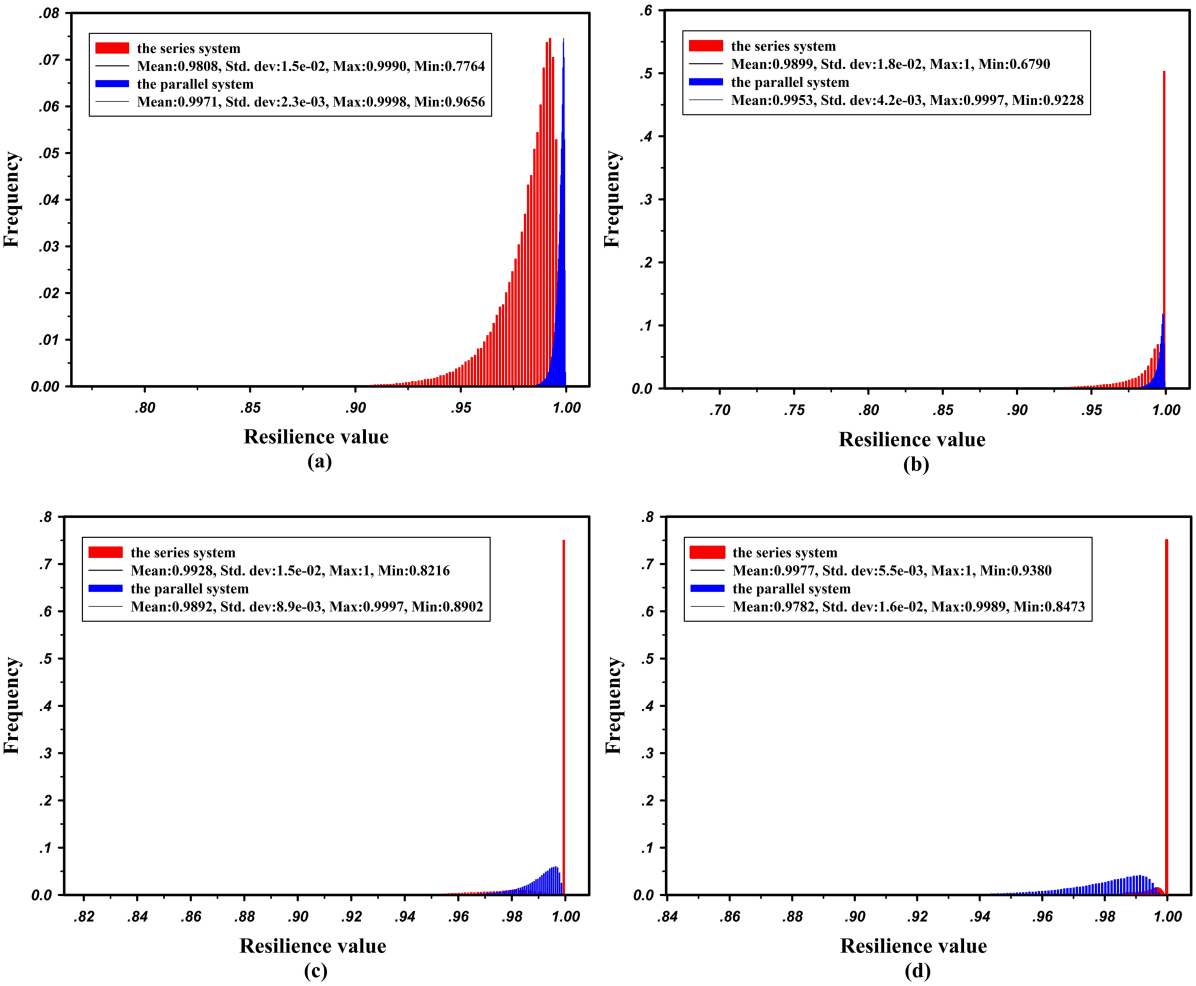
Comparison of empirical pdfs of resilience for series and parallel systems under capacity degradation of different components. **(a)** Com_1_, **(b)** Com_2_, **(c)** Com_3_, and **(d)** Com_4_.

From Table 2, one can see the effects of the same component performance degradation on different system structures. The empirical resilience probability distribution functions (pdfs) of the series and parallel systems under each component’s degradation are represented in Fig 4. When Com_1_ suffers a disruption, the maximum flow of both systems will decline, and the recovery time equals that of Com_1_ because any capacity degradation on Com_1_ will cause the maximum flow of the two systems to drop. The same capacity decrease on Com_1_ will cause greater performance degradation for the series system because the capacity of Com_1_ equals the maximum flow of the series system, while it provides only a part of flow for the parallel system. Hence, the mean resilience of the parallel system under disruptions on Com_1_ is larger than that of the series system, as shown in Fig 4(a). When a disruption occurs at Com_2_, Com_3_ or Com_4_, the capacity degradation on such components may not affect the maximum flow of the series system, and once the maximum flow drops, the recovery time of the system will be shorter than that of the component. This phenomenon occurs because these components are not the bottlenecks of the system maximum flow and have some capacity redundancy. If the component capacity drops and the system performance is not affected, the system has high robustness to withstand such disruption. Conversely, such disruptions must cause a decrease of the maximum flow on the parallel system, and the recovery time of the system equals that of the component. Similarly, the capacity degradation of Com_2_ with the smaller initial capacity will also result in a larger percentage of performance degradation for the series system. Consequently, the mean resilience value of the parallel system under disruptions on Com_2_ is also larger than that of the series system, as shown in Fig 4(b), although a case exists in which the capacity degradation on Com_2_ does not cause maximum flow degradation for the series system. Nevertheless, as shown in Fig 4(c) and 4(d), the mean resilience values of the series system under disruptions on Com_3_ and Com_4_ are higher than the parallel ones. On the one hand, the probability that the maximum flow of the system is not affected by the capacity degradation on Com_3_ and Com_4_ is high for the series system. On the other hand, the percentage of performance degradation of the parallel system caused by the capacity degradation on Com_3_ and Com_4_ is higher than that in the series case because Com_3_ and Com_4_ with higher initial capacity provide the most flow for the parallel system.

**(2) Resilience analysis along with increasing number of components**

For the two types of structures, consider that the number of components increases gradually (indeed, the number can be increased to infinity). In addition, all components are identical, i.e., each component shares the same initial capacity and disruption probability and follows the same capacity degradation distribution and recovery time distribution. The corresponding parameters are provided in Table 3.

**Table 3:** The parameter data of the case.

Initial capacity	Remaining capacity	Probability	Recovery time
4	0*	0.1	*t* ∼ *lnN*(0.3, 0.5^2^)
	0.8	0.2	
	2	0.3	
	3.2	0.4	

* *Pr*{the remaining capacity of the component is 0} = 0.1.

**Fig 5:**
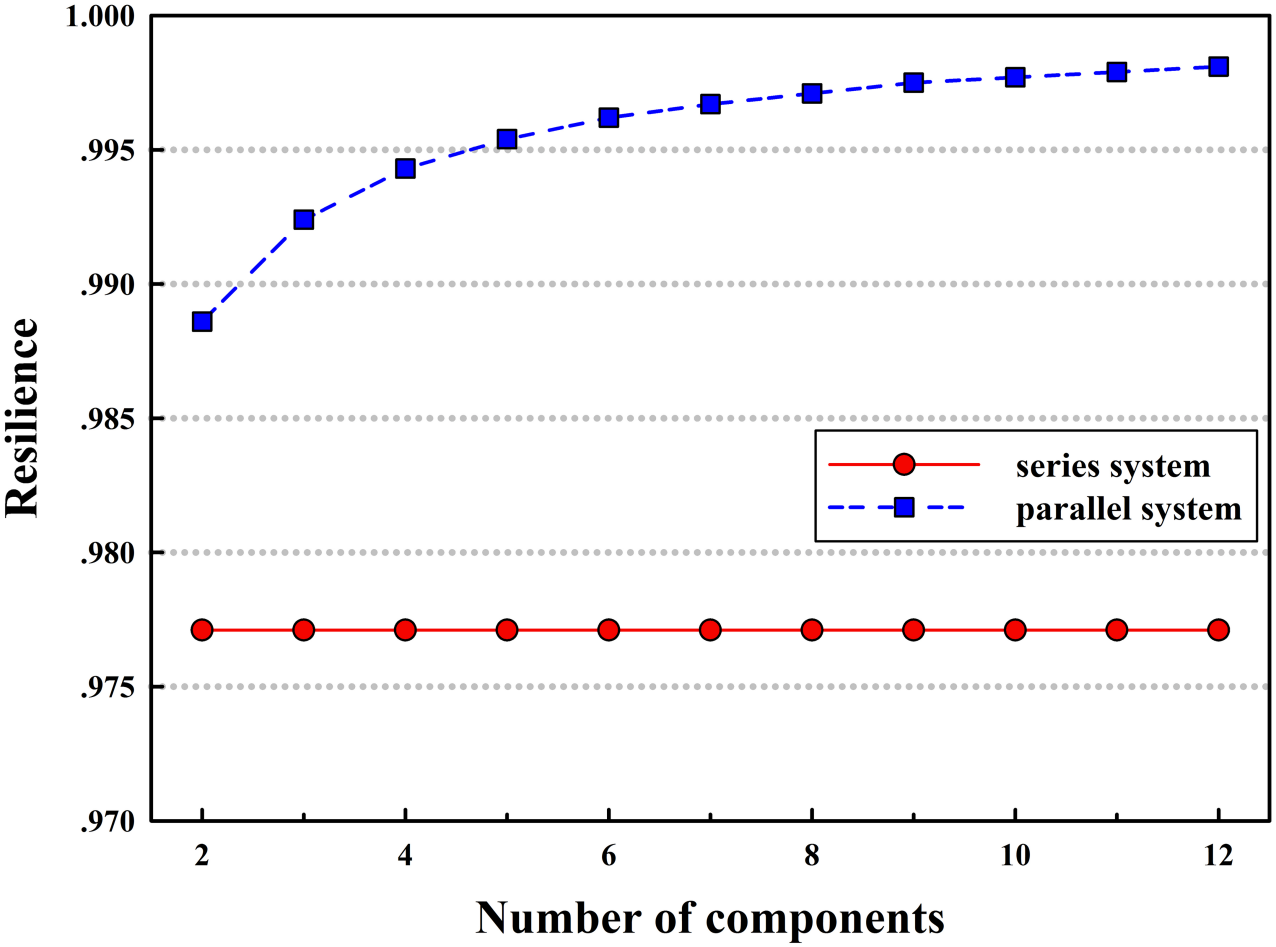
System resilience curves with increasing components.

Using our analytic system resilience models, the expected maximum flow-based resilience of series and parallel systems with increasing number of components can be calculated as in Fig 5. One can see that the expected maximum flow-based resilience of parallel systems increases with increasing number of components and that of series systems remains constant. This occurs mainly because the maximum flow of the parallel system increases along with that of the components, and the impact of the capacity degradation of one component decreases with increasing system maximum flow, eventually leading to increased expected system resilience. However, for the series system, the increase of components will not cause the variation of the system maximum flow, so the expected system resilience is constant. Note that with the same components, the expected maximum flow-based resilience of the parallel system is always larger than that of the series system because the former features higher capacity redundancy in the face of a disruptive event.

### Networked systems

#### Illustrated example

**Fig 6:**
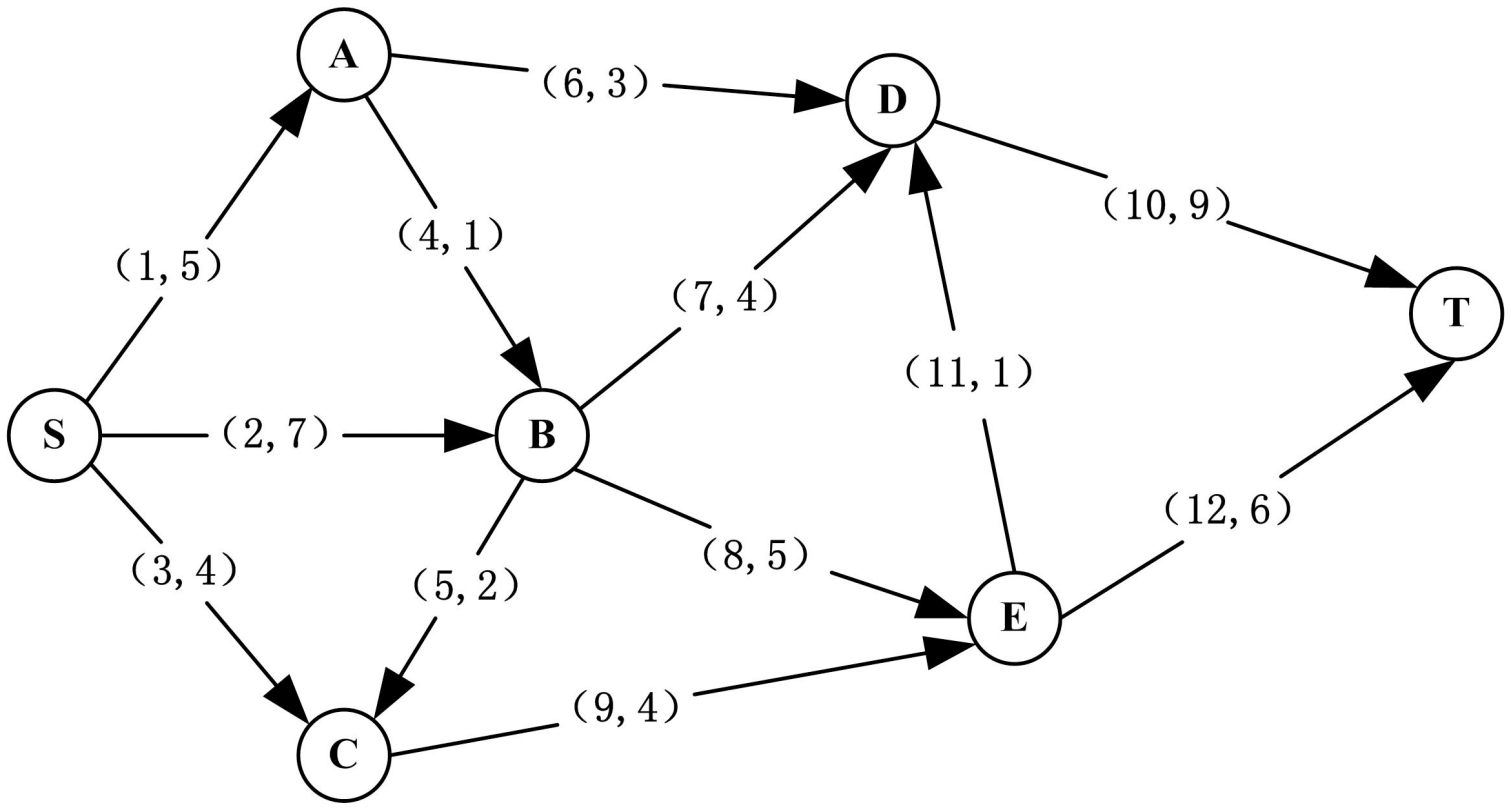
Illustrative road network example.

In our network case study, a road network of Seervada Park was used as the illustrated example. The Seervada Park Problem was used by Hillier and Lieberman [65] as an example to discuss the shortest path, the minimum spanning tree and the maximum flow problems in Operations Management. Henry and Ramirez-Marquez [37] then used this problem to analyze the network resilience, in which Seervada Park was located in hilly terrain where a river runs through it, and two disruptive events (a rock slide and a flood) can cause damage on different road segments. In our paper, we used the road network topology and the maximum daily capacity of each road segment provided by Henry and Ramirez-Marquez [37] and assumed the parameters of the disruption, the capacity degradation and the recovery time of components. The road network has 12 links as shown in Fig 6, and the link labels represent their index number and capacity. The quantitative approach is also applicable to any other networked system that is similar to the road network used here.

As previously discussed, the resilience of a system is computed with the restoration process of performance. To compute the resilience of the road network, we use the same disruptive events provided in [37] and assume a new one as follows:

**Disruption** 1: a river that runs through the entrance of the road network floods; as a result, one of the road segments, Com_1_-Com_5_, will be destroyed;

**Disruption** 2: a rockslide occurs in the center of the road network, which will result in the destruction of one of the road segments, Com_6_-Com_9_;

**Disruption** 3: snow covers the end of the road network, which will lead to the traffic control of one of the road segments, Com_10_-Com_12_.

Here, the disruption probability of road segments can be seen in column 3 of Table 4, and their capacity degradation follows discrete distributions as shown in columns 4–5. Because the segments under the same type of disruption use the same resource for restoration, three lognormal distributions are used to reflect different restoration rapidities under different disruptions as illustrated in column 6. In most instances, flooding takes the most time to restore, the rockslide requires the second most time, and snow cover recovers most quickly. In addition, we consider T* = 10 time units as the strict upper bound of the recovery time. In this case, only three types of disruptions are considered. Nevertheless, the resilience analysis method for networks in the section of Methodology is a general one, and it can be used to analyze the network resilience under different types of disruptions given the corresponding disruption probability, capacity degradation distribution and recovery time distribution.

**Table 4:** The parameter data of the case.

Component	Disruptive event	Disruption probability	Remaining capacity	Probability	Recovery time
Com_1_	Disruption 1	0.15	0*	0.2	*t*_1−5_ ∼ *lnN*(1.5, 1^2^)
			1	0.3	
			2.5	0.5	
Com_2_		0.1	0	0.2	
			1.4	0.3	
			3.5	0.5	
Com_3_		0.15	0	0.2	
			0.8	0.3	
			2	0.5	
Com_4_		0.05	0	0.2	
			0.2	0.3	
			0.5	0.5	
Com_5_		0.05	0	0.2	
			0.4	0.3	
			1	0.5	
Com_6_	Disruption 2	0.1	0	0.1	*t*_6−9_ ∼ *lnN*(1, 0.5^2^)
			0.6	0.2	
			1.5	0.4	
			1.8	0.1	
			2.4	0.2	
Com_7_		0.05	0	0.1	
			0.8	0.2	
			2	0.4	
			2.4	0.1	
			3.2	0.2	
Com_8_		0.05	0	0.1	
			1	0.2	
			2.5	0.4	
			3	0.1	
			4	0.2	
Com_9_		0.1	0	0.1	
			0.8	0.2	
			2	0.4	
			2.4	0.1	
			3.2	0.2	
Com_10_	Disruption 3	0.05	4.5	0.5	*t*_10−12_ ∼ *LN*(0.5, 0.3^2^)
			5.4	0.3	
			7.2	0.2	
Com_11_		0.1	0.5	0.5	
			0.6	0.3	
			0.8	0.2	
Com_12_		0.05	3	0.5	
			3.6	0.3	
			4.8	0.2	

* *Pr*{the remaining capacity of Com_1_ is 0} = 0.2.

#### Resilience analysis and discussion

**Fig 7:**
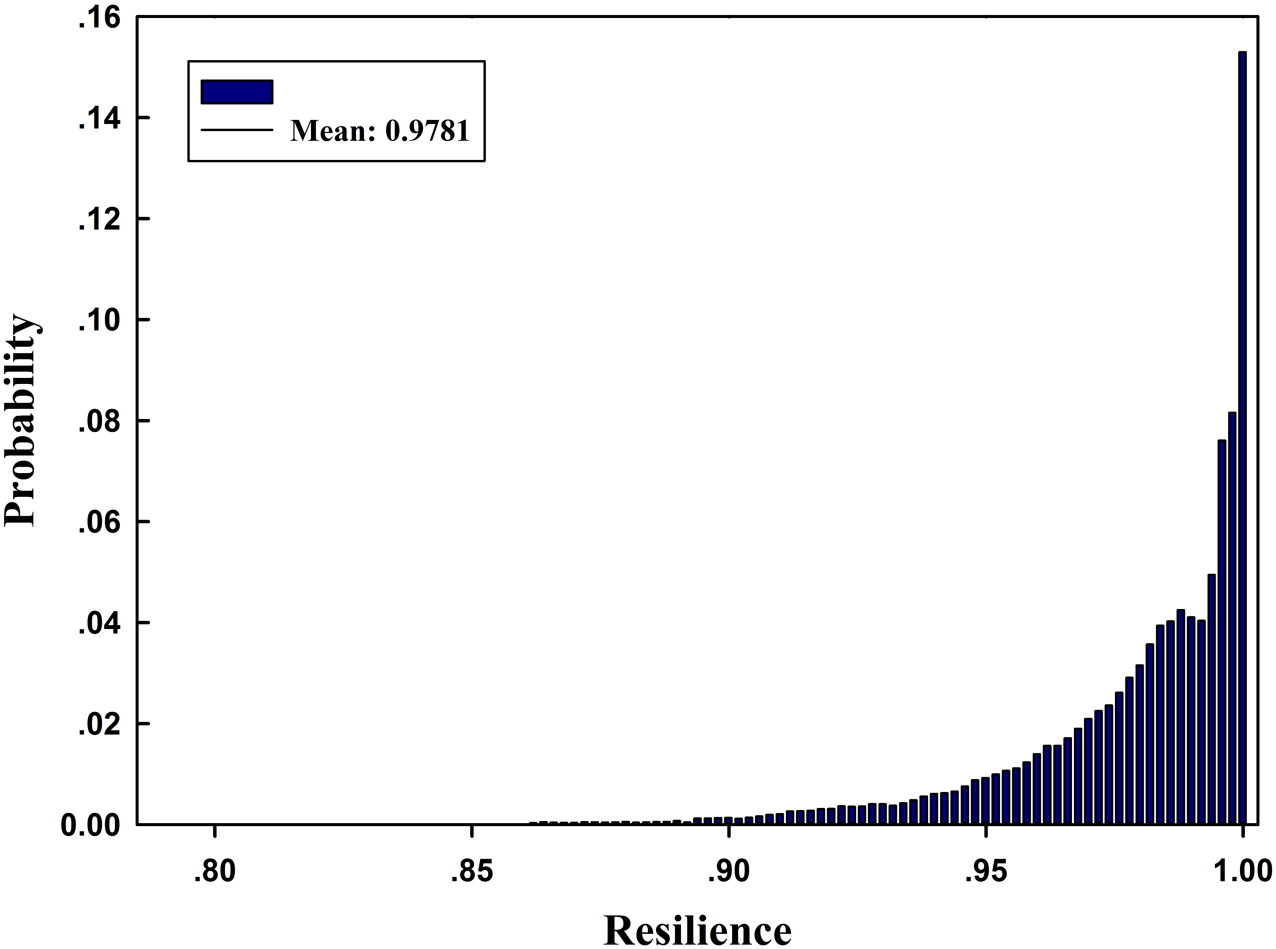
Pdf of maximum flow-based network resilience.

Under normal conditions, the network can handle a maximum flow of 14 units. The disruptive event leads to capacity degradation on component *i*, which may cause degradation of the system maximum flow. Using our Monte Carlo-based simulation method, the empirical resilience of the networked system can be obtained as $\bar{R_{S}}=0.9781$ after 10^5^ iterations. The pdf of the resilience is illustrated in Fig 7. One can see that the probability that the maximum flow-based resilience is greater than 0.975 is over 60%. This shows that the network resilience is very high under most disruptions, and it also has some probability to be small under certain disruptions.

**Fig 8:**
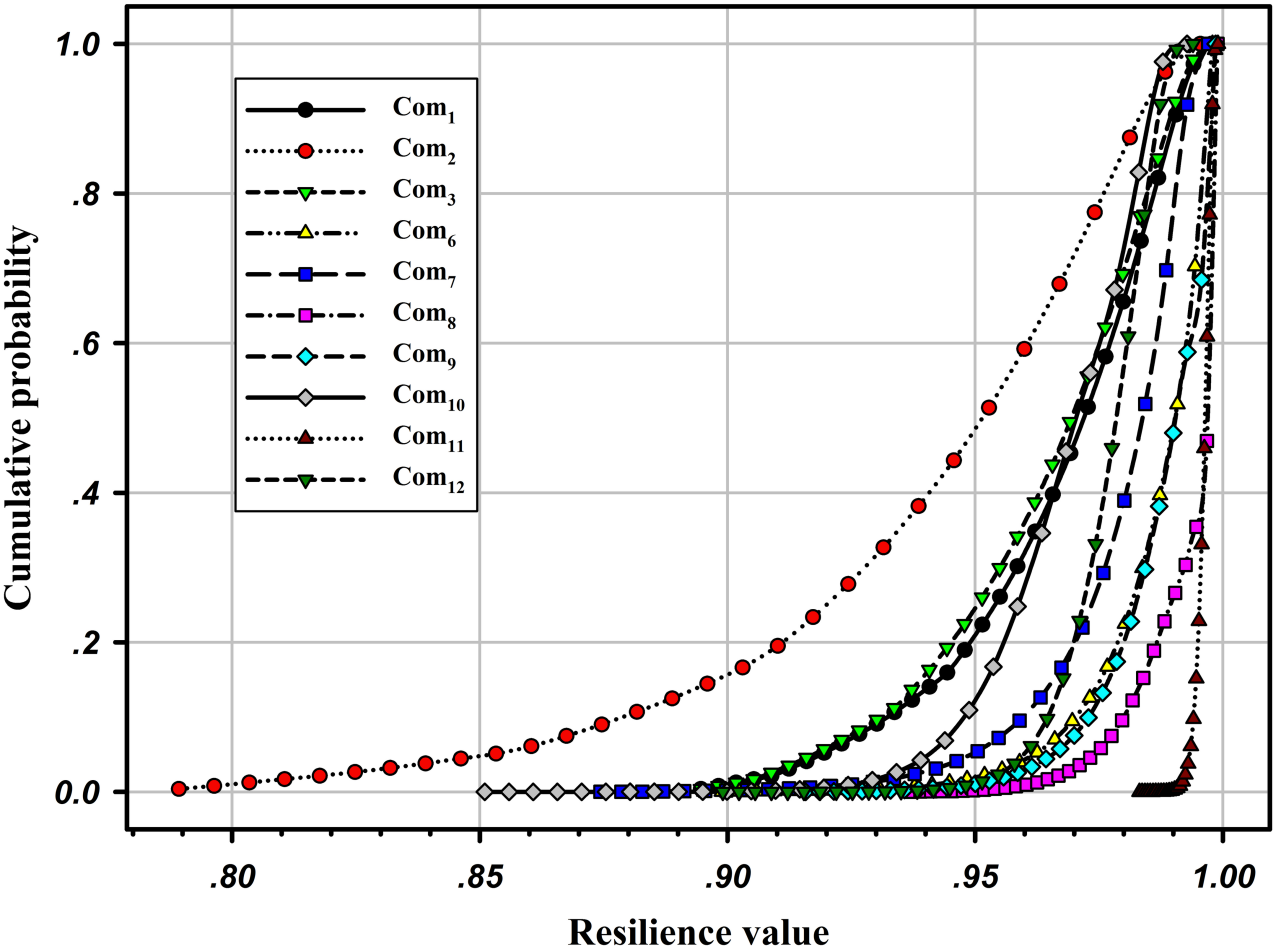
Cdfs of the network resilience due to disruptions on different components.

Meanwhile, Fig 8 illustrates the cumulative probability distributions (cdfs) of network resilience under disruptions of different components. In Fig 8, only the system resilience caused by disruptions on Com_2_ may be less than 0.85, and it also has the widest bound. In other words, a disruption that occurs on Com_2_ has the most adverse effect on the maximum flow-based resilience of the road network. In contrast, the network resilience based on all other components is greater than 0.85. Note that for Com_4_ and Com_5_, their capacity degradation has no effect on the entire network; i.e., the system maximum flow will not degrade even if the capacity of the two components drops to 0, so there are no corresponding curves for the two components shown in Fig 8. The effect of different components on the network may also change along with the target system resilience. For example, the curve for Com_12_ is below that of Com_7_ if R<0.97 (i.e., the network is more resilient if the capacity degradation occurs on Com_7_), while this behavior changes if R>0.97, and the network becomes more resilient if the capacity degradation occurs on Com_12_.

**Fig 9:**
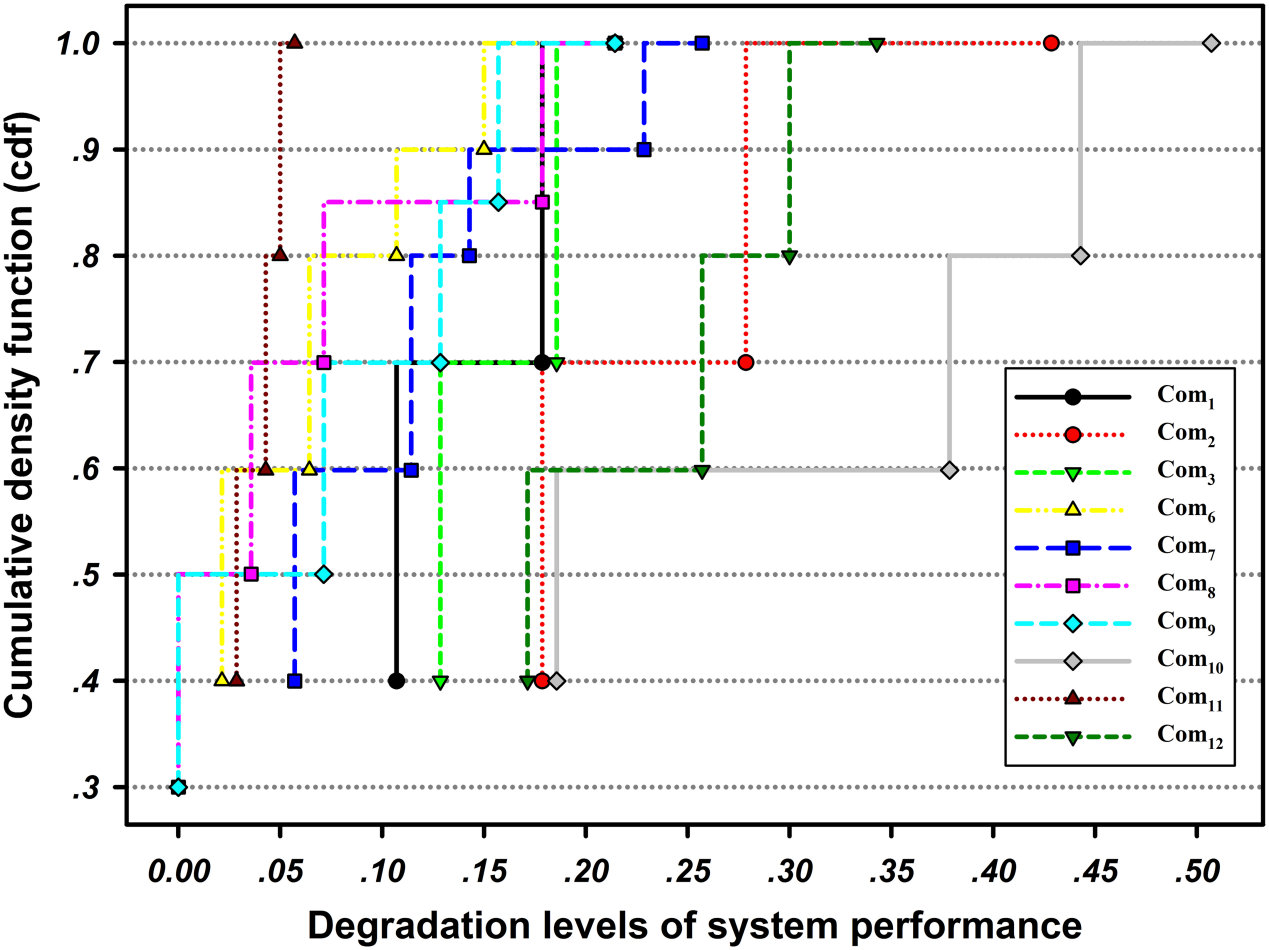
Network performance degradation levels of each component expressed in cdfs.

**Fig 10:**
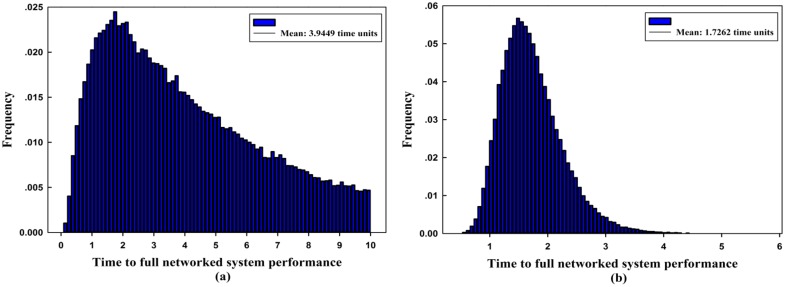
Time to full recovery in histogram (approximate pdf) form under the capacity degradation of (a) Com_2_ and (b) Com_10_.

The degradation levels of the network maximum flow caused by different capacity reductions in each component are plotted in Fig 9. It can be seen that Com_11_ incurs the smallest mean performance degradation of the network, i.e., the system can withstand most disruptions on Com_11_. Consequently, when a disruption occurs on Com_11_, the network has the thinnest resilience bound, as shown in Fig 8. Moreover, taking Com_2_ and Com_10_ as examples, the degradation levels of system performance caused by Com_10_ are larger than that of Com_2_; however, the network is more resilient under the disruption of Com_10_ in Fig 8. This occurs mainly because of the different system recovery times of the two. Fig 10 depicts the pdfs of system recovery time when the capacity degradation occurs on Com_2_ and Com_10_. The comparison illustrates that the maximum recovery time is 9.9998 time units for Com_2_ and 5.7755 time units for Com_10_. Meanwhile, the mean recovery time of the latter is 1.7262 time units, which is much smaller than the former one of 3.9449 time units, so the latter can have a higher probability to recover in a shorter time interval. In this paper, we assume that only one disruption can occur at a time, and the possible combinations of disruptions will be studied in our future work. In this case, if multiple disruptive events occur simultaneously, more than one road segments’ capacity may be degraded, possibly causing greater maximum flow degradation and longer system recovery time.

**Fig 11:**
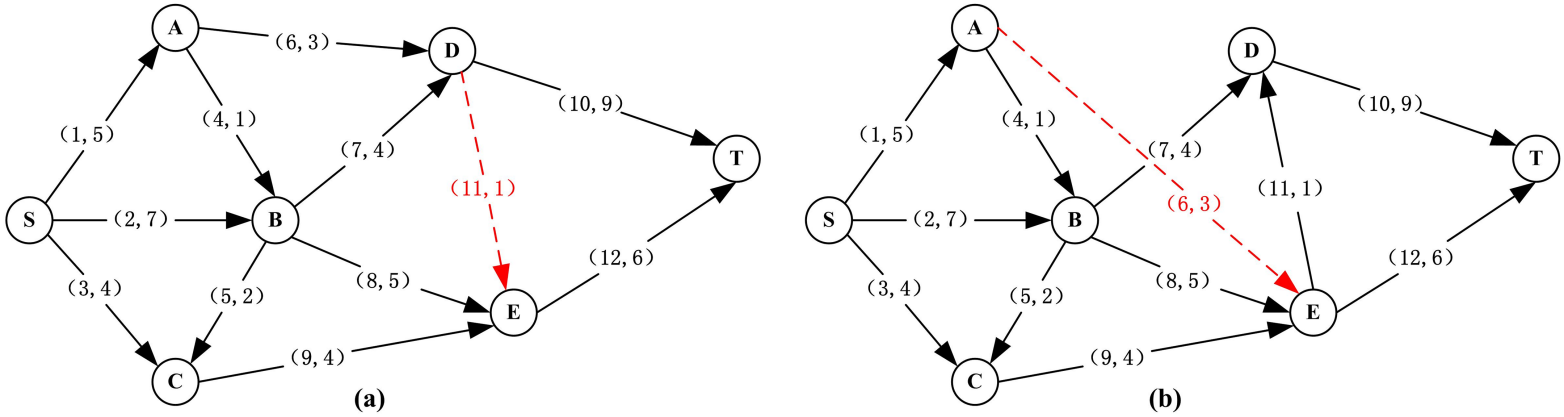
Two more topologies of the road network. **(a)** Topology_2_ and **(b)** Topology_3_.

It is obvious that the network resilience differs for different topologies. In our case study, we use two more topologies for comparison with the original topology in Fig 6 (Topology_1_), and the two topologies (Topology_2_ and Topology_3_) are illustrated in Fig 11. Here, the two topologies can handle a maximum flow of 13 and 11 units under normal operation, respectively. Hence, with the same links and nodes, the three topologies have different capacity redundancy, where Topology_1_ has the minimum redundancy, Topology_3_ has the maximum redundancy, and Topology_2_ is between the two. Here, the capacity redundancy is considered as the ratio of the total spare capacity to the total working capacity [66].

**Fig 12:**
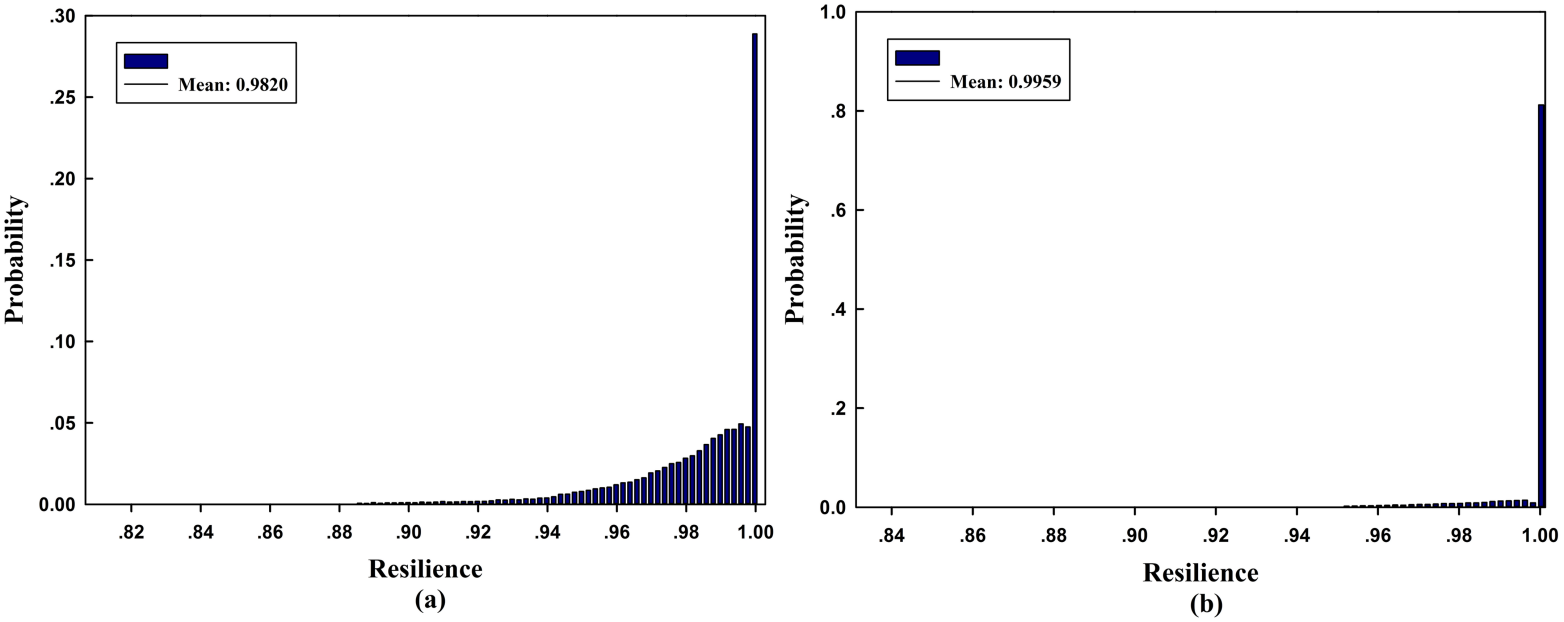
Pdfs of maximum flow-based network resilience for the two topologies. **(a)** Topology_2_ and **(b)** Topology_3_.

The pdfs of network resilience for Topology_2_ and Topology_3_ are shown in Fig 12. Comparing the resilience distributions for the three topologies, one can see that the topology with higher capacity redundancy has higher empirical resilience, i.e., $\bar{R_{T_{3}}}>\bar{R_{T_{2}}}>\bar{R_{T_{1}}}$. This phenomenon occurs because the probability to migrate the network flow on the component under disruption to other redundant ones is larger in the network with higher redundancy, so such capacity degradation on components has no effect on the network maximum flow. In this case, one can see that the network topology affects the system resilience. Nowadays, it’s really an essential and challenge issue to answer how to cope with those unidentified threats, and non-stationarity or evolving hazards. When these threats and hazards are identified, it is useful to apply our resilience measurement framework to compare and select better structure/topology and recovery strategy for the system.

**Fig 13:**
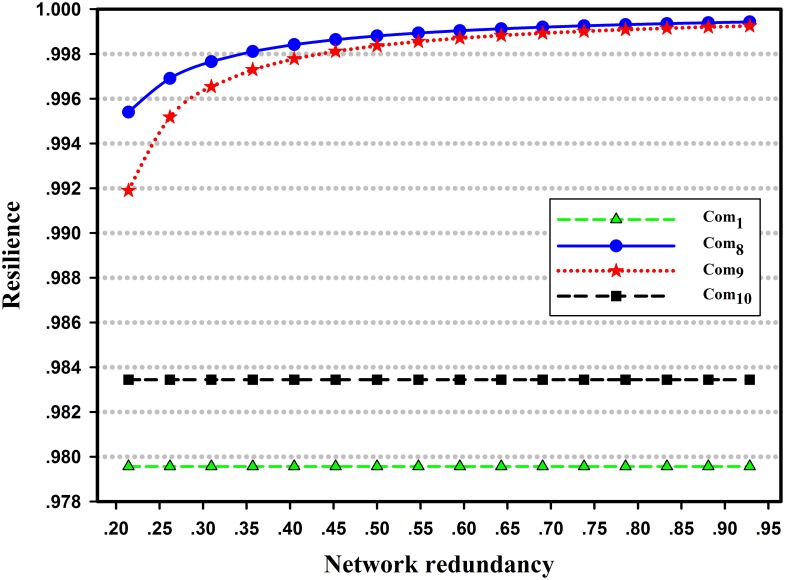
Comparison of network resilience with the increase of capacity redundancy on different components.

However, not all systems with more capacity redundancy are more resilient. To discuss how capacity redundancy affects the system resilience, the capacities of Com_1_, Com_8_, Com_9_ and Com_10_ in the Seervada Park Problem are increased as examples, as these capacity increases will not change the maximum flow of the network. As shown in Fig 13, the network resilience increases with increasing capacity redundancy on Com_8_ or Com_9_, while it remains constant if the capacity redundancy is located at Com_1_ or Com_10_. This phenomenon occurs because the capacity degradation of components follows discrete distributions. For degraded Com_8_ and Com_9_, once their capacities are recovered to 3 and 3.2, respectively, the maximum flow of the network will be fully recovered. The capacity increases on these two components will decrease the recovery time of the network. The greater the capacity redundancy the two components has, the faster the system can recover, so the network resilience increases with increasing capacity redundancy on Com_8_ and Com_9_. For Com_1_ and Com_10_, there is no level of capacity degradation that can support the initial network maximum flow. If we increase the capacity of Com_1_ or Com_10_, the maximum flow of the network will still not be recovered until the component is fully restored, i.e., the network recovery time will not change along with the capacity redundancy on Com_1_ or Com_10_, so the network resilience remains constant.

In summary, not all capacity redundancy can improve the system resilience, and even if the system resilience is improved, the effectiveness is different. Thus, it is important to choose the proper location for increased redundancy.

## Conclusion

This paper focuses on modeling maximum flow-based system resilience according to the resilience of components, which was always neglected in previous engineering research. This type of model can be used not only to evaluate the system resilience but also to help system structure decision making.

For this purpose, this paper proposes two new component-based system resilience models for series and parallel systems, in which the maximum flow is used as the key performance index (KPI). Using the models, the expected system resilience can be calculated for series and parallel systems, and a Monte Carlo-based simulation is also provided to verify the correctness and effectiveness of our models and analyze the resilience of a more complex system with a network structure. In fact, the methods we used to calculate the system resilience are almost the same for series and parallel systems and real networks. The only difference is that the maximum flow of series and parallel systems can be calculated easily, while no explicit function exists for networks. Thus, we build theoretical maximum flow-based system resilience models for series and parallel systems and use Monte Carlo-based simulation to explore the network resilience. The resilience of a real network cannot be inferred by comparison with either a series or parallel system, but in our future work, we will use the Monte Carlo-based simulation method proposed in this paper to study how the network resilience changes along with the topology, the scale, the degree, the distance, etc. In addition, the system resilience result is strongly correlated with the distributions of component recovery time, which determines the recovery time of the system. In our paper, we use only the most widely used distribution—i.e., a lognormal distribution—to describe the component recovery time. If the recovery time of components follows other types of distributions, the method proposed in our paper can be easily extended. For series and parallel systems, we only need to modify the expectation expression of component recovery time in the analytic system resilience models. For the networked system, we only need to change the distribution of the component recovery time in the simulation method. In our paper, the maximum flow of the system is determined by both the capacity of components and the performance relationship between the system and components: (1) for a series system, the maximum flow of the system is determined by the minimum capacity of its components, i.e., $C_{S}=\min_{i=1,2,\ldots ,n}\left\{C_{i}\right\}$; (2) for a parallel system, the system’s maximum flow is formed by adding all capacities of its components, i.e., $C_{P}=\sum_{i=1}^{n}C_{i}$; and (3) for a networked system, the maximum flow of the system is defined as the amount of flow passing from the source to the sink, and the algorithm provided by Edmonds and Karp [64] is used to calculated the system maximum flow according to the network topology and the capacities of components. The analytic resilience models for series and parallel systems and the simulation method for networked systems are derived and designed based on the performance combination of the system mentioned above, i.e., how the performance of the components contributes to that of the system. Thus, although we consider only the maximum flow as our system performance metric in this paper, both the system resilience models and simulation method can also be used for systems with the same type of performance relationship between components and system. For example, the equivalent spring constant for a parallel spring is determined by the spring constant of its component—i.e., $K_{P}=\sum_{i=1}^{n}\left(K_{i}\right)$—and the equivalent spring constant-based resilience can be calculated using our maximum flow-based resilience model for the parallel system; the total resistance of resistors connected in series is the sum of their individual resistance values—i.e., $R_{S}=\sum_{i=1}^{n}\left(R_{i}\right)$—and the total resistance can also be calculated using our resilience model for the parallel system, although the resistors are laid out in a series structure.

Finally, according to the calculated results of series and parallel systems based on resilience models and the simulation results of the networked system, three general conclusions are derived: (1) two analytic maximum flow-based resilience models for series and parallel systems are derived, and the resilience of the system with the corresponding performance structures can be calculated directly by using the two analytic models given the distributions of performance degradation and recovery time for components; (2) for systems with identical components, the resilience of the parallel system increases with increasing number of components, while it remains constant in the series system; and (3) a system with redundant performance is usually more resilient than one without redundant performance. However, not all redundant capacities of components can improve the system resilience, the effectiveness of the capacity redundancy depends on where the redundant capacity is located.

In this work, the analytic resilience models provided by us do not consider the relationship between the recovery time and the severity of the disruption. However, in some situations, the recovery time depends on the severity of the disruption; further research can be performed to study this problem. In addition, given all the probabilities that a component’s failure will trigger the failure of successive components and the corresponding distributions of capacity degradations and recovery time, the Monte Carlo simulation method proposed in our paper can still be used to calculate the maximum flow degradation caused by the cascading failures and the recovery time of the network under each iteration, and then the system resilience can be estimated. Therefore, our research method is extendable to systems with cascading failures. These topics could be valuable avenues to explore in future extensions of this study.
